# Molecular manipulation of keratin 8/18 intermediate filaments: modulators of FAS-mediated death signaling in human ovarian granulosa tumor cells

**DOI:** 10.1186/s13048-016-0217-z

**Published:** 2016-02-24

**Authors:** Sarah K. Trisdale, Nicolette M. Schwab, Xiaoying Hou, John S. Davis, David H. Townson

**Affiliations:** Corning Life Sciences, Woburn, MA 01801 USA; Department of Molecular, Cellular and Biomedical Sciences, University of New Hampshire, Durham, NH 03824 USA; Veterans Affairs Medical Center and Olson Center for Women’s Health, University of Nebraska Medical Center, Omaha, NE 68198 USA; Current address: Department of Animal & Veterinary Sciences, University of Vermont, Burlington, VT 05405 USA

**Keywords:** Granulosa cells, Granulosa cell tumor, Keratin filaments, Sex cord tumor, Apoptosis, FAS

## Abstract

**Background:**

Granulosa cell tumors (GCT) are a rare ovarian neoplasm but prognosis is poor following recurrence. Keratin intermediate filaments expressed in these tumors are a diagnostic marker, yet paradoxically, may also constitute a target for therapeutic intervention. In the current study, we evaluated keratin 8/18 (K8/18) filament expression as a mechanism of resistance to apoptosis in GCT, specifically focusing on regulation of the cell surface death receptor, Fas (FAS).

**Methods:**

The GCT cell line, KGN, was transiently transfected with siRNA to *KRT8* and *KRT18* to reduce K8/18 filament expression. Expression of K8/18, FAS, and apoptotic proteins (PARP, cleaved PARP) were evaluated by fluorescence microscopy, flow cytometric analysis, and immunoblotting, respectively. The incidence of FAS-mediated apoptosis in KGN cells was measured by caspase 3/7 activity. All experiments were performed independently three to six times, using a fresh aliquot of KGN cells for each experiment. Quantitative data were analyzed by one- or two-way analysis of variance (ANOVA), followed by a Tukey’s post-test for multiple comparisons; differences among means were considered statistically significant at *P* < 0.05.

**Results:**

Control cultures of KGN cells exhibited abundant K8/18 filament expression (~90 % of cells), and minimal expression of FAS (<25 % of cells). These cells were resistant to FAS-activating antibody (FasAb)-induced apoptosis, as determined by detection of cleaved PARP and measurement of caspase 3/7 activity. Conversely, siRNA-mediated knock-down of K8/18 filament expression enhanced FAS expression (> 70 % of cells) and facilitated FasAb-induced apoptosis, evident by increased caspase 3/7 activity (*P* < 0.05). Additional experiments revealed that inhibition of protein synthesis, but not MEK1/2 or PI3K signaling, also prompted FasAb-induced apoptosis.

**Conclusions:**

The results demonstrated that K8/18 filaments provide resistance to apoptosis in GCT by impairing FAS expression. The abundance of keratin filaments in these cells and their role in apoptotic resistance provides a greater mechanistic understanding of ovarian tumorgenicity, specifically GCT, as well as a clinically-relevant target for potential therapeutic intervention.

## Background

Granulosa cell tumors (GCT) represent approximately 5 % of ovarian malignancies, yet constitute the most prevalent sex cord-stromal ovarian neoplasms [1, 2]. Prognosis is favorable when detected early, but recurrent GCT may be life-threatening. The rarity of these tumors and their low potential for malignancy (i.e., slow growth over a prolonged period) has reduced the urgency to study and develop standard treatment regimens for these neoplasms compared to other ovarian cancers. However, GCT have a high rate of reappearance following resection [2–4], and such recurrence often leads to late detection and poor prognosis [5, 6]. Distinct from other ovarian cancers, GCT are endocrine tumors [7]. High estrogen secretion by GCT can lead to a variety of malignancies and pathological states, including oligomenorrhea, hirsutism, breast cancer, endometrial hyperplasia and adenocarcinoma [5, 8–10]. Additionally, GCT have many morphological and biochemical features akin to granulosa cells of mature, preovulatory follicles (e.g., FSH-responsive and steroid hormone production) [11–14].

Tumorigenicity of GCT is linked to a somatic missense mutation in the Forkhead Box L2 (*FOXL2*) gene (c.402C→G; p.C134W). The mutation occurs in nearly all adult-type GCT, yet is absent in juvenile GCT [14], indicating the *FOXL2* mutation is etiologically significant in the development of adult GCT. The function of the mutated *FOXL2* is not fully understood, however it is postulated to be a tumor suppressor. Overexpression of *FOXL2* induces expression of cell death receptors of the Tumor Necrosis Factor Receptor Superfamily, particularly the cell surface death receptor, Fas (FAS, also known as TNFRSF6), which facilitates apoptosis of ovarian granulosa cells [15, 16]. In contrast, granulosa cells expressing the *FOXL2* C134W mutant lack these death receptors and are resistant to apoptosis [16]. In spite of recent attempts to develop therapeutic approaches using genetic manipulation in mouse models [14, 17, 18] and transcriptomic analysis to identify candidate driver genes [19, 20], relatively little is known about selectively treating GCT.

Current treatment of GCT entails surgical resection of the ovary and/or platinum-based chemotherapy - originally developed to specifically eliminate ovarian surface epithelial (OSE) tumors [21]. However, ovarian carcinomas, including GCT and OSE tumors, share the common trait of expressing keratin intermediate filaments. Keratin type I cytoskeletal 18 protein (K18, also known as *KRT18*), in particular, is expressed in malignant cells and is a diagnostic marker to delineate morphologic heterogeneity and neoplastic changes [22, 23]. Others argue that the prevalence of intermediate filaments (i.e., keratins, vimentin, and desmin) in GCT, as well as other ovarian carcinomas, limits their value in differential diagnosis among these groups [24].

The well-understood function of keratin intermediate filaments is to provide stability and morphological integrity to cells, yet in recent years a more dynamic role has emerged. Structurally, keratin filaments are heterotetramers of two type I and two type II keratins. Keratin 8/18 (K8/K18) intermediate filaments, for example, are composed of keratin type II cytoskeletal 8 protein and type I cytoskeletal 18 protein. Originally named for their mechanical stability, K8/18 filaments are “stress filaments” that provide resistance against sheer stress; but they also influence intracellular signaling mechanisms, counteract physiological stressors, enhance wound healing, and prevent apoptosis [25, 26]. Notably, K8/18-deficient epithelial cells are sensitive to physiological stressors, including cytokine-induced apoptosis [27–30]. In this context, K8/18 filaments within cells associate with the tumor necrosis factor receptor 1-associated death domain (TRADD) protein [31]. TRADD is a critical intracellular intermediate to TNFR1-induced death signaling, which in turn interacts with the Fas-Associated Death Domain (FADD) protein [32]. By sequestering TRADD, K8/18 filaments potentially attenuate and influence interactions between FAS, FADD and other downstream apoptotic molecues, thus providing resistance to cytokine-mediated apoptosis. In carcinoma-derived cell lines (HeLa, HepG2, KLE), for example, disruption of K8/18 filament expression sensitizes the cells to cisplatin-induced apoptosis by increasing FAS expression, but also by decreasing the expression of the anti-apoptotic protein, cellular FLICE inhibitory protein (c-FLIP, also known as CFLAR) [33]. Based upon these observations, keratin filaments constitute a plausible target for immune-based approaches to cancer treatment, and might provide an alternative to chemotherapy of GCT, in particular.

The granulosa-like KGN cell line, established from an adult GCT, has provided insight about the molecular and cellular features of GCT, along with potential vision for treatment [34]. For instance, a recent investigation revealed that, in contrast to OSE-derived cancers, inhibition of NF-κB does not sensitize KGN cells to TRAIL- or cisplatin-induced apoptosis [35]. Interestingly, human granulosa cells and KGN cells share a similar resistance to other forms of immune-mediated death, including FAS-induced apoptosis [34, 36, 37]. Acknowledging that K8/18 filament loss in other epithelial cell types leads to increased FAS expression and enhanced vulnerability to FAS-mediated apoptosis [28, 33], we hypothesized in the current study, that K8/18 filaments in KGN cells provide a similar mechanism of influence. Our objectives were to first determine if KGN cells express K8/18 filaments as seen in granulosa cells, GCT, and OSE tumors [22, 23, 30] and then to identify the cellular mechanism(s) by which K8/18 filaments potentially influence resistance to FAS-mediated apoptosis. We observed that, indeed, KGN cells express an abundance of K8/18 filaments and genetic knockdown of K8/18 results in enhanced expression of FAS and increased vulnerability to FAS-mediated apoptosis.

## Methods

### Cells and culture conditions

The human granulosa cell tumor line, KGN, was generously provided by Dr. Fukuzawa (RIKEN Cell Bank, Koyadai, Japan). KGN cells were maintained in DMEM/F12 supplemented with 10 % FBS at 37 °C with 5 % CO_2_, 95 % air and 95 % humidity. Cells were seeded in multi-well plates, T25-flasks (Corning, Corning, NY) or microchamber slides (Nunc, Rochester, NY) at a relative seeding density of 5 × 10^4^ cells/mL and grown to 70 % confluency prior to treatment.

### Immunofluorescent visualization of K8/K18 intermediate filaments

The K18 protein is known to dimerize with K8 protein to form K8/K18 filaments in ovarian steroidogenic cells [30, 38]. KGN cells grown on coverslips were washed with PBS, then fixed with ice-cold 4 % paraformaldehyde (PFA) in PBS (pH 7.4) for 10 min. The fixed cells were permeabilized for 10 min in 0.4 % Triton-X-100 in PBS. Cells were washed and blocked with blocking buffer (0.2 % Triton-X-100 in PBS with 10 % normal donkey serum) for 30 min at room temperature. Cells were labeled with mouse anti-human K18-FITC-conjugated antibody (CY90; Sigma-Aldrich, St. Louis, MO) diluted 1:100 in blocking buffer for 3.5 h at room temperature. Cells were washed again and probed with Rhodamine Phalloidin (1:1500, Life Technologies, Grand Island, NY) and 4′,6-diamidino-2-phenylindole (DAPI; 30 nM) in blocking buffer for 30 min at room temperature. For the negative control, KGN cells were also exposed to a mouse anti- human IgG-FITC-conjugated antibody (Sigma-Aldrich, St. Louis, MO) diluted 1: 100 in blocking buffer in place of the primary. The coverslips were washed and then mounted with Fluoromount-G® (Southern Biotechnology Associates, Inc, (Birmingham, AL). Images were captured using a Zeiss 710 Meta Confocal Laser Scanning Microscope and analyzed using the Zeiss Zen 2010 software (Carl Zeiss Microscopy, LLC, Thornwood, NY, USA).

### Flow cytometric quantification of FAS surface and K18 expression

Cultured cells were trypsinized using Cellgro 0.25 % Trypsin-EDTA (Corning, Corning, NY) and fixed with 2 % PFA in microtubule-stabilizing buffer. For FAS staining, the cells were stored in PFA; for K18 and total FAS staining, the fixed cells were permeabilized in 70 % ethanol. For antibody labeling, both types of cell preparations were washed with PBS + 0.1 % BSA, and then either labeled overnight with mouse anti-human FAS (CH11; EMD Millipore, Darmstadt, Germany) antibody diluted to 20 μg/mL in 10 % normal goat serum (NGS) + PBS + 1 % BSA, or for 1 h with mouse anti-human K18-FITC-conjugated antibody (Sigma-Aldrich, St. Louis, MO) diluted 1:100 in PBS + 1 % BSA. Separate fixed cell suspensions were labeled with mouse anti-human IgG-FITC-conjugated antibody (Sigma-Aldrich, St. Louis, MO) to serve as the negative control. For FAS staining, the cells were subsequently washed with PBS + 0.1 % BSA, and incubated with Alexa Fluor® 488 (Life Technologies, Grand Island, NY) diluted 1:200 in 10 % NGS + PBS + 1 % BSA. All samples were washed with PBS + 0.1 % BSA and analyzed using a 4 color, dual laser FACS Calibur Flow Cytometer (BD Biosciences, San Jose, CA), quantifying 10,000 cells for each sample. Data were collected using CellQuest software (BD Biosciences; San Jose, CA) and then analyzed using WinMDI (Joe Trotter; Purdue University, West Lafayette, IN) to quantify FAS and keratin filament expression.

### Induction of FAS-mediated apoptosis

KGN cells were pretreated with cycloheximide (CHX; 0.25 μg/mL; Sigma-Aldrich, St. Louis, MO), the MEK1/2 inhibitor PD98059 (30 μM; Cell Signaling Technologies, Danvers, MA) or the PI3K inhibitor Wortmannin (100 nM; EMD Millipore, Darmstadt, Germany) for 2 h in serum-free culture medium. After pretreatment, the cells were exposed to FAS-activating antibody (FasAb; 1 μg/mL; clone CH11; EMD Millipore, Darmstadt, Germany), or staurosporine (1 μM; MP Biomedical, Santa Ana, CA) as a positive control, to induce apoptosis. The cells were exposed to the above treatments for 8 and 24 h, at which time caspase 3/7 activity was measured by the Caspase-Glo® 3/7 Assay and cell viability was determined using the CellTiter 96® AQueous One Solution Cell Proliferation Assay (MTS), respectively. Assays were conducted according to the manufacturer’s instructions (Promega, Madison, WI).

### Immunoblot analysis for FAS, cFLIP, cleaved PARP and β-actin

Nearly confluent cells from the above-described experiments were washed with PBS and harvested in lysis buffer (10 mM Tris–HCl; 1 mM EDTA; 1 mM EGTA; 100 mM NaCl; 1 % Triton X-100; 0.5 % Nonidet P-40, pH 7.4) containing a cocktail of kinase, protease and phosphatase inhibitors (Sigma-Alrich, St. Louis, MO). Cells were scraped, collected, sonicated and then resuspended in 2X SDS loading buffer (100 mM Tris-Cl, pH 6.8 + 4 % SDS, 0.2 % bromophenol blue, 20 % glycerol, 200 mM DTT) before denaturing at 95 °C for 5 min. Total cellular proteins were separated by SDS-PAGE and transferred to polyvinylidene difluoride (PVDF) membranes (EMD Millipore,Darmstadt, Germany). Immunoblotting was performed using antibodies to detect human cFLIP (also known as CFLAR) (rabbit anti-human CFLAR; Sigma Aldrich, St. Louis, MO) and human FAS (clone C-20; Santa Cruz Biotechnology, Santa Cruz, CA). The membranes were stripped and reprobed for cleaved human poly ADP ribose polymerase (PARP) (# 9542, Cell Signaling Technology, Danvers, MA), to verify the apoptotic effect, and β-actin (clone AC-15; Sigma Aldrich, St. Louis, MO) to ensure equivalent protein loading.

### Short interfering RNA (siRNA) knockdown of KRT8/18 gene transcription

KGN cells were transiently transfected with 10 pmol siRNA constructs to *KRT8* and *KRT18* (Smartpool Accell *KRT8* and *KRT18* siRNA; Dharmacon RNAi, GE Healthcare, Lafayette, CO). Transfection was achieved using Lipofectamine™ RNAiMAX in OptiMEM® Reduced Serum Media for final 100 nM RNAi duplexes according to the manufacturer’s instructions (Life Technoloies, Grand Island, NY). Cells were grown to 70 % confluency then switched to antibiotic-free DMEM/F12 + 10 % FBS before siRNA- Lipofectamine™ duplexes were introduced. The cells were also exposed to Lipofectamine™ and a non-targeting siRNA (si*CTL*; Silencer® Select Negative Control #1; Ambion Inc., Foster City, CA) as negative controls. Knock-down of *KRT8/18* expression was evaluated by immunofluorescence as described above.

Knock-down of *KRT8/18* was also evaulated using an in-cell western assay according to the manufacturer’s instructions (LI-COR®, Lincoln, NE). Following a 72 h exposure to siRNA, the cells were washed with PBS, then fixed and permeabilized in 100 % MeOH. Detection of K18 and β-Actin (internal control) expression was achieved using an antibody cocktail of mouse anti-human K18 (CY90; Sigma-Aldrich, St. Louis, MO) and rabbit anti-human β-Actin (13E5, Cell Signaling Technolgy, Danvers, MA) followed by a secondary antibody cocktail (goat anti-mouse IgG H + L DyLight 800 and goat anti-rabbit IgG H + L DyLight 680, Cell Signaling Technology, Danvers MA). The cells were imaged using a LI-COR® Odyssey® Classic Infrared Imaging scanner. Staining intensity for K18 was normalized to the staining intensity for β-actin using the provided software.

### Statistical analysis

All experiments were independently replicated three to six times, using a fresh aliquot of KGN cells (passage 23-27) to initiate each experiment. Data were analyzed by one- or two-way analysis of variance (ANOVA), followed by a Tukey’s post-test for multiple comparisons; differences among means were considered statistically significant at *P* < 0.05.

## Results

### Keratin and β-actin expression in KGN cells

**Fig. 1:**
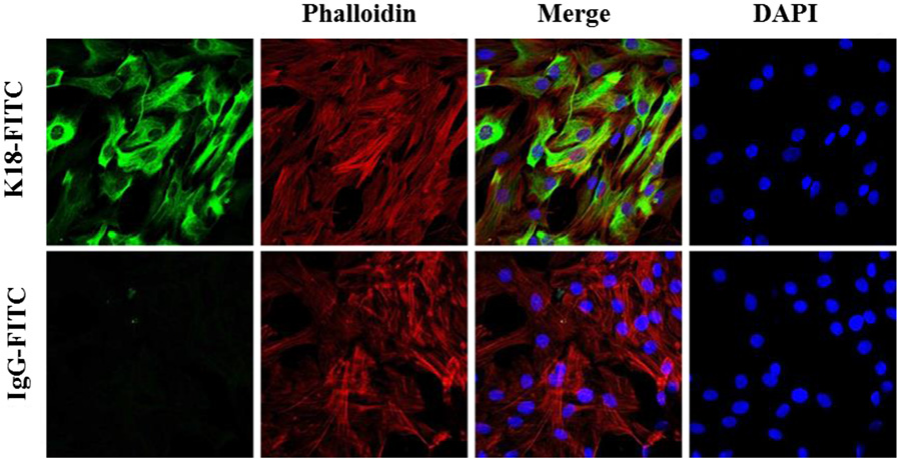
Representative images of immunofluorescent staining of K8/18 and β-actin filaments in cultured KGN cells. Cells were labeled with mouse anti-human K18-FITC-conjugated antibody (keratin filaments, green fluorescence), Phalloidin (β-actin filaments, red fluorescence), DAPI (nuclei, blue fluorescence) or mouse anti-human IgG-FITC-conjugated antibody (negative control). Individual and merged images of fluorescence are shown

Immunofluorescent staining of cultured KGN cells revealed an abundance of K8/18 filament expression throughout the cells (Fig. 1, green fluorescence). Both cytoplasmic and perinuclear expression was evident, generally in parallel with β-actin filament expression (Fig. 1, red fluorescence). Thus, both K8/18 and β-actin filament expression are prominent cytoskeletal components of cultured KGN cells.

### Abundant K18 expression but minimal FAS expression on KGN Cells

**Fig. 2:**
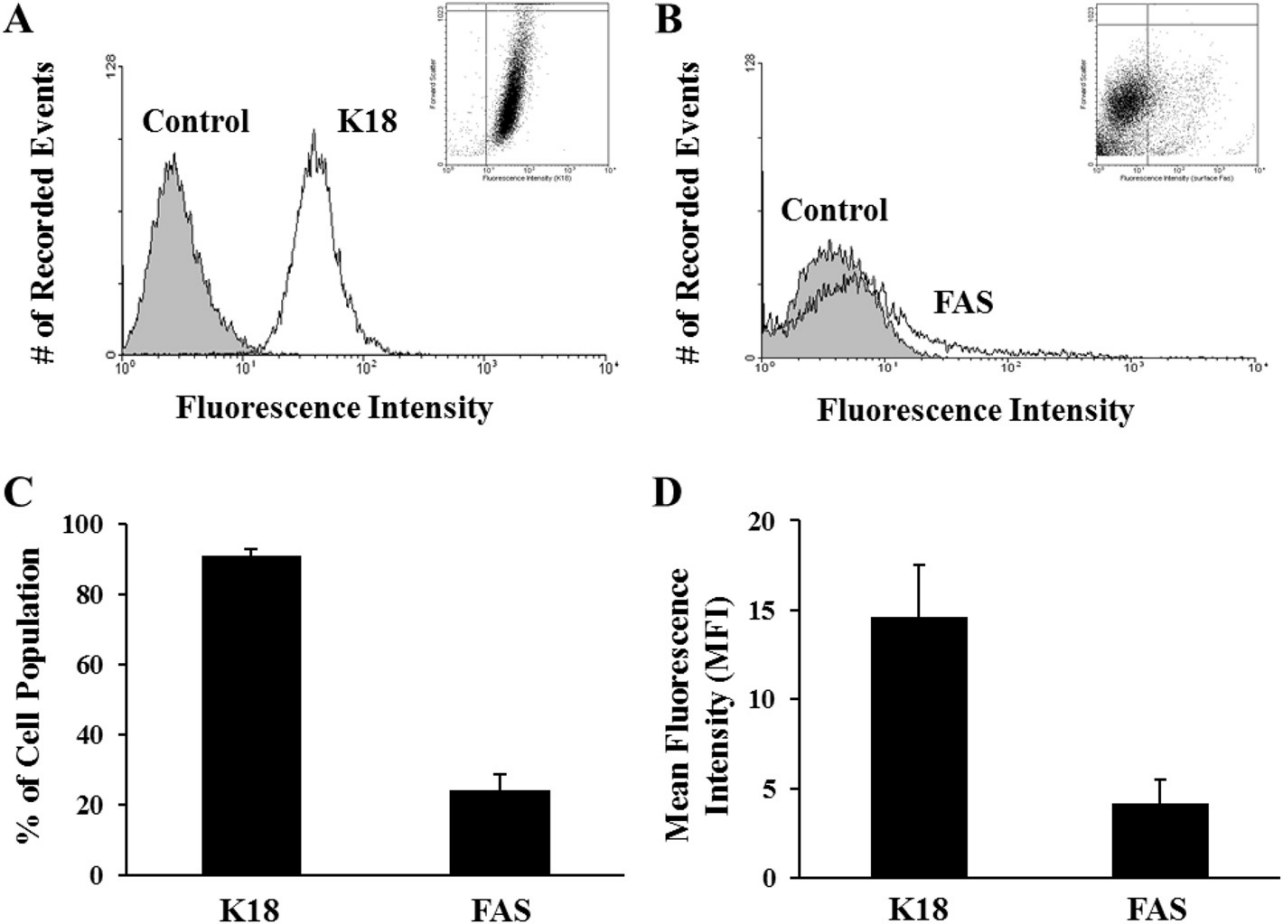
Flow cytometric analysis of K18 and FAS expression by cultured KGN cells. **a**, **b** Representative histograms and dot plots of fluorescence intensity vs. number of recorded events for (**a**) K18 expression and (**b**) cell surface expression of FAS; **c** Percentage of KGN cell population (± SEM) stained positively for K18 and FAS; **d** Mean fluorescence intensity (± SEM) of K18 and FAS expression by KGN cells. Bar graphs represent three independent experiments

Flow cytometric analysis demonstrated ~ 91 % of KGN cultured cells expressed K18 (Fig. 2a and c); consistent with the abundance of keratin filament staining observed by immunofluorescence (representative image shown in Fig. 1). In contrast, only 24 % of the cells expressed FAS (Fig. 2b and c), indicative of an overall inverse relationship between K8/18 filament abundance and FAS expression. Mean fluorescent intensity measures (an indication of fluorescence per cell) were similarly high for keratin filament expression, but low for FAS expression (Fig. 2d).

### Inhibition of protein synthesis sensitizes KGN cells to FasAb-induced apoptosis

**Fig. 3:**
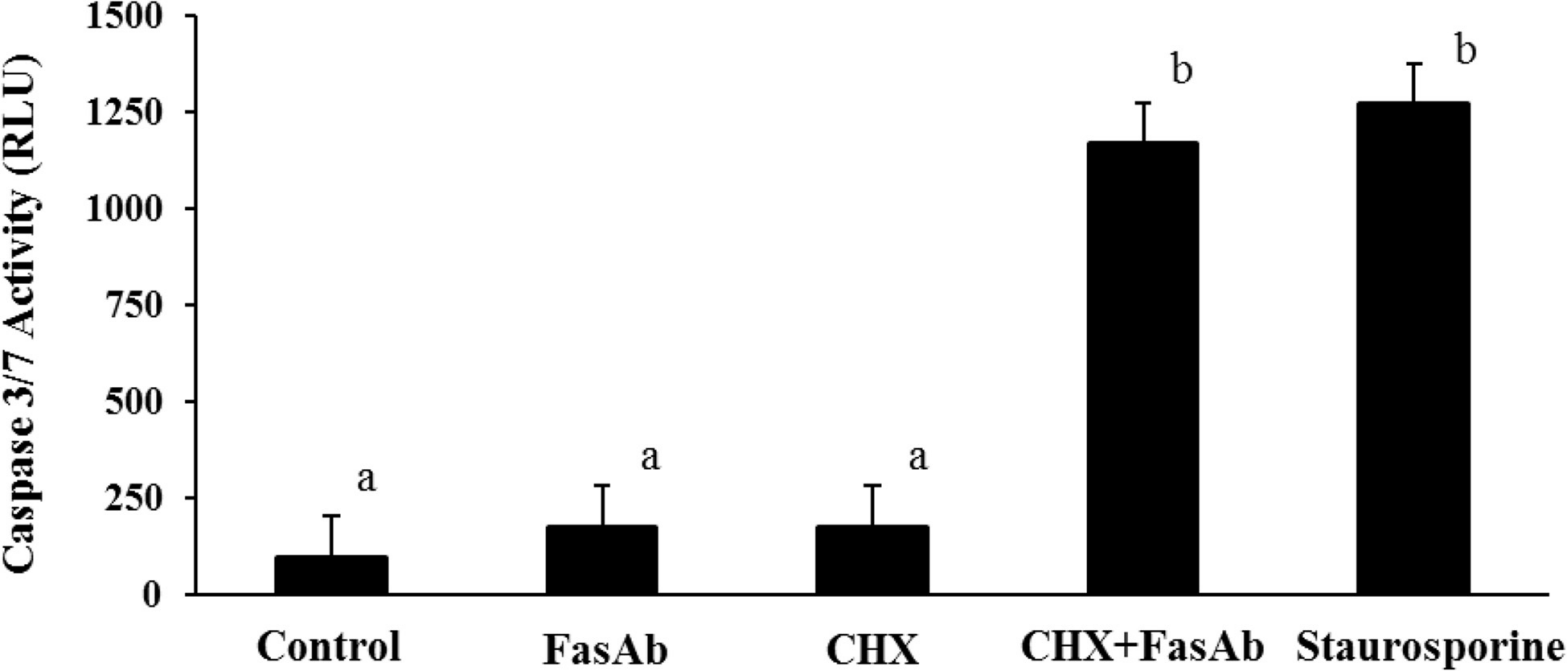
FasAb -induced apoptosis in cultured KGN cells, quantified by caspase 3/7 activity (RLU ± SEM) following inhibition of *de novo *protein synthesis. The cultures were exposed to cycloheximide (0.25 μg/mL; CHX), for a total of 10 h and FasAb (1 μg/mL) during the last 8 h of culture. Staurosporine treatment (1 μM) was included as the positive experimental control (*n* = 6 independent experiments; different letters denote differences, *P* < 0.05)

Exposure of KGN cells to FasAb for 8 h failed to induce apoptosis, as measured by caspase 3/7 activity (Fig. 3). Conversely, pretreatment with cycloheximide (CHX, 0.25 μg/ml), a protein synthesis inhibitor, augmented FasAb-induced apoptosis (*P* < 0.05; Fig. 3). The effect was similar to that of Staurosporine (1 μM), a chemotherapeutic agent used as a positive control (*P* < 0.05, Fig. 3). Exposure of KGN cells to CHX alone had no effect (*P* > 0.05). Similarly, disruption of MEK1/2 (via PD98059; 30 μM) and PI3K (via Wortmannin; 100 nM) pro-survival pathways in KGN cells, failed to augment FasAb-induced apoptosis (data not shown).

### FAS and cFLIP expression following FasAb-induced apoptosis in KGN cells

**Fig. 4:**
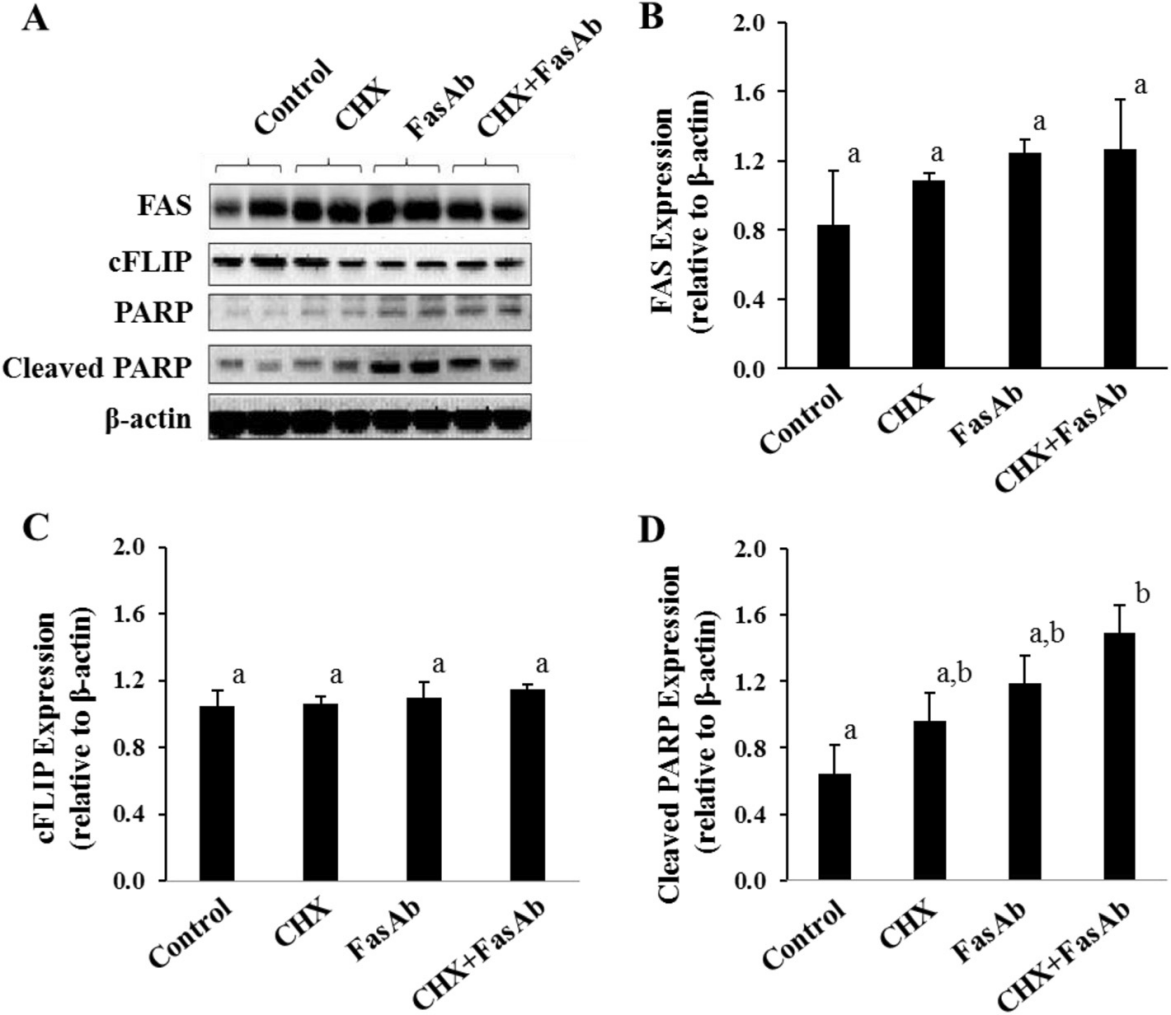
Immunodetection of FAS, cFLIP, PARP, cleaved PARP and β-actin following exposure to the protein synthesis inhibitor CHX (0.25 μg/mL) and FasAb (1 μg/mL) in KGN cells. **a** Representative immunoblot of FAS, cFLIP, PARP, cleaved PARP and β-actin following treatments; **b** Relative expression of FAS normalized to β-actin (means ± SEM) following treatment; **c** Relative expression of cFLIP normalized to β-actin (means ± SEM) following treatment; **d** Relative expression of cleaved PARP normalized to β-actin (Means ± SEM) following treatment. (*n* = 3 experiments, different letters denote differences, *P* < 0.05)

Immunoblotting revealed no overt changes in FAS expression following exposure to CHX or FasAb in cultured KGN cells (*P* > 0.05, Fig. 4a and b). Similarly, there was no effect of CHX and/or FasAb on the expression of cFLIP (*P* > 0.05, Fig. 4a and c). In contrast, effects of these treatments were evident by the detection of PARP and cleaved PARP (Fig. 4a and d), but only the combined treatment led to a doubling (*P* < 0.05) of cleaved PARP (Fig. 4d). The results confirmed the combined apoptotic effects of CHX and FasAb as shown in Fig. 3.

### siRNA knockdown of KRT8 and KRT18 enhances FasAb-induced apoptosis of KGN cells

**Fig. 5:**
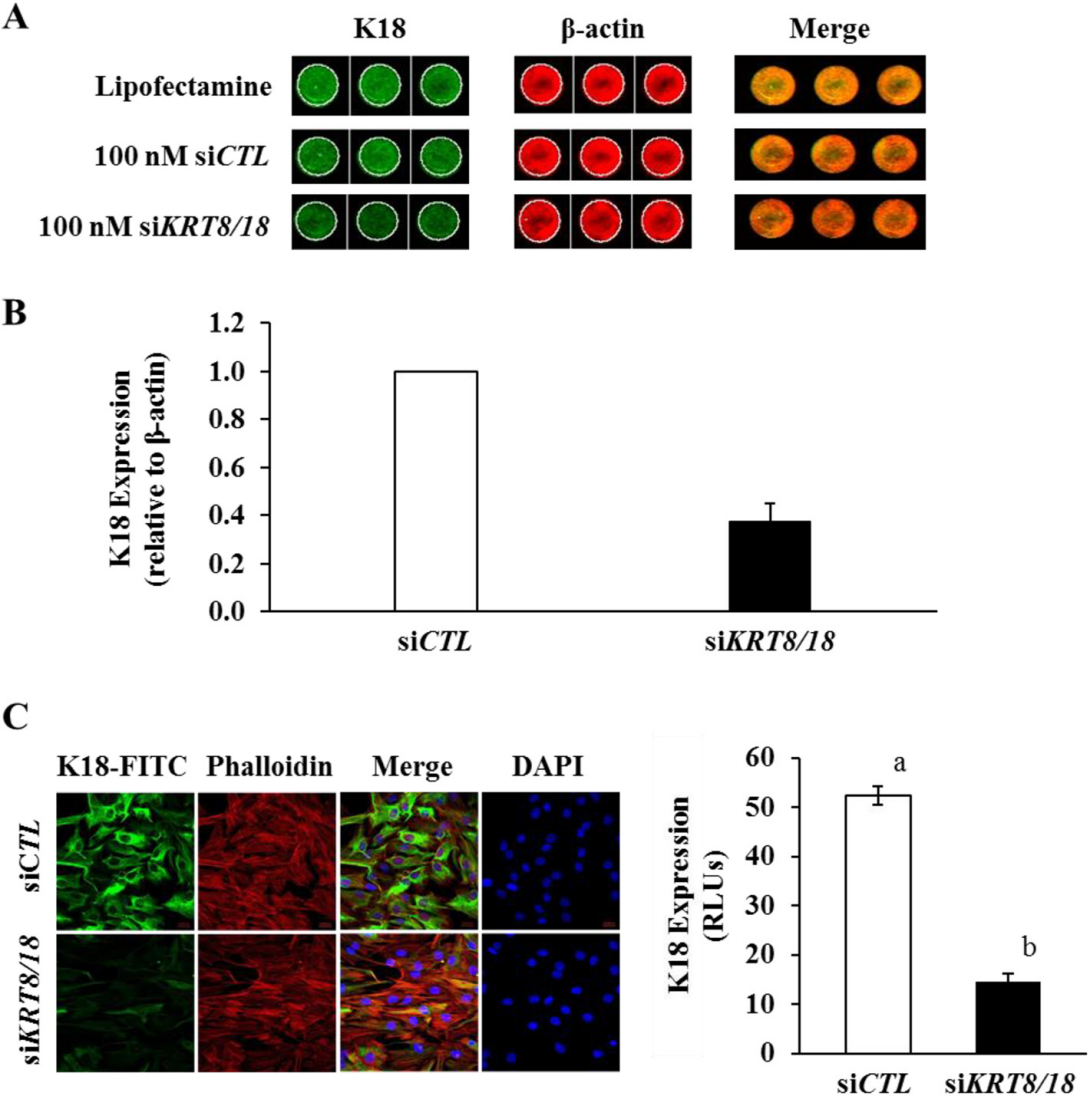
Immunodetection of K18 and β-actin protein expression in KGN cells mock transfected (si*CTL*) or transfected with 100 nM siRNA to *KRT8* and *KRT18*. **a** Representative in-cell western dual detection of K18 protein (green), β-actin protein (red), and combined (merged image-yellow) following exposure to either Lipofectamine™ or equimolar concentrations of a non-targeting siRNA (si*CTL*) as controls, or 100 nM siRNA to *KRT8/18* (si*KRT8/18*). Diminished expression of K18 in the single and merged images was evident following 72 h exposure to si*KRT8/18*; **b** Quantification of K18/β-actin expression compared to si*CTL* following si*KRT8/18* transfection. The mean (± SEM) following treatment for three independent, replicate experiments is depicted. **c** Representative image of immunofluorescent staining of K18 and actin filaments in cultured KGN cells. Keratin (K18) filament expression stained with FITC (green); β-actin filament expression stained with Phalloidin (red); nuclei stained with DAPI (blue); and merged image of FITC, Phalloidin and DAPI. Bar graph represents quantification of K18 fluorescence (mean relative light units, RLU ± SEM). (*n* = 6 independent images; different letters denote differences, *P* < 0.05)

**Fig. 6:**
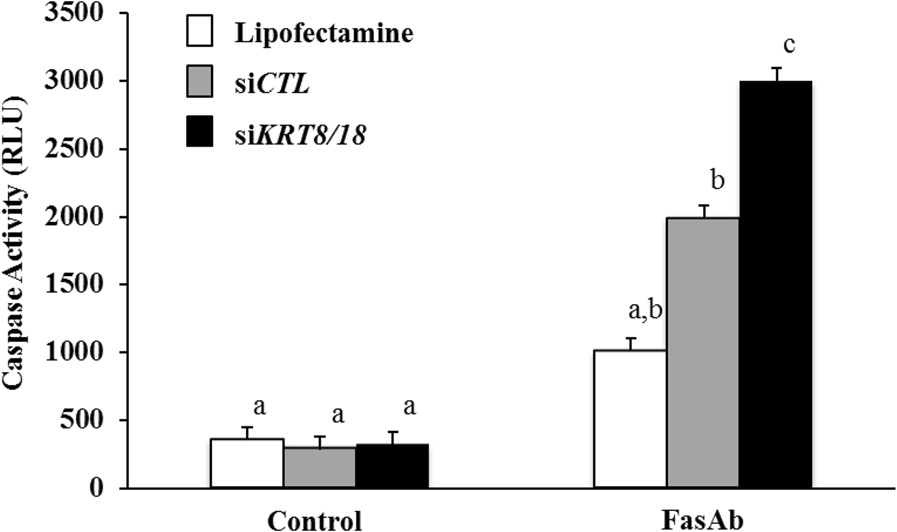
FasAb-induced apoptosis as measured by caspase 3/7 activity (RLU ± SEM) following siRNA transfection with si*CTL* and si*KRT8/18* in cultured KGN cells (*n* = 3 experiments; different letters denote differences, *P* < 0.05)

Knockdown of *KRT8* and *KRT18* in KGN cells (siRNA) reduced keratin expression by 63 % as determined by in-cell western (Fig. 5a and b) and 72 % by confocal imaging (Fig. 5c). Subsequent experiments evaluated the effect of *KRT8* and *KRT18* knockdown on FasAb-induced apoptosis in KGN cells. Treatment with Lipofectamine™, non-targeting siRNA (si*CTL*) or *KRT8/18* siRNA alone had no effect on caspase 3/7 activity (*P* > 0.05, Fig. 6); however, subsequent exposure to FasAb for 8 h resulted in a higher incidence of apoptosis in *KRT8/18* siRNA-treated cells compared to controls (*P* < 0.05; Fig. 6). Of interest, treatment with Lipofectamine™ and si*CTL* moderately augmented the sensitivity of KGN cells to the apoptosis-inducing effects of FasAb (Fig. 6).

### Down-regulation of keratin filaments (siRNA) enhances expression of FAS on KGN cells

**Fig. 7:**
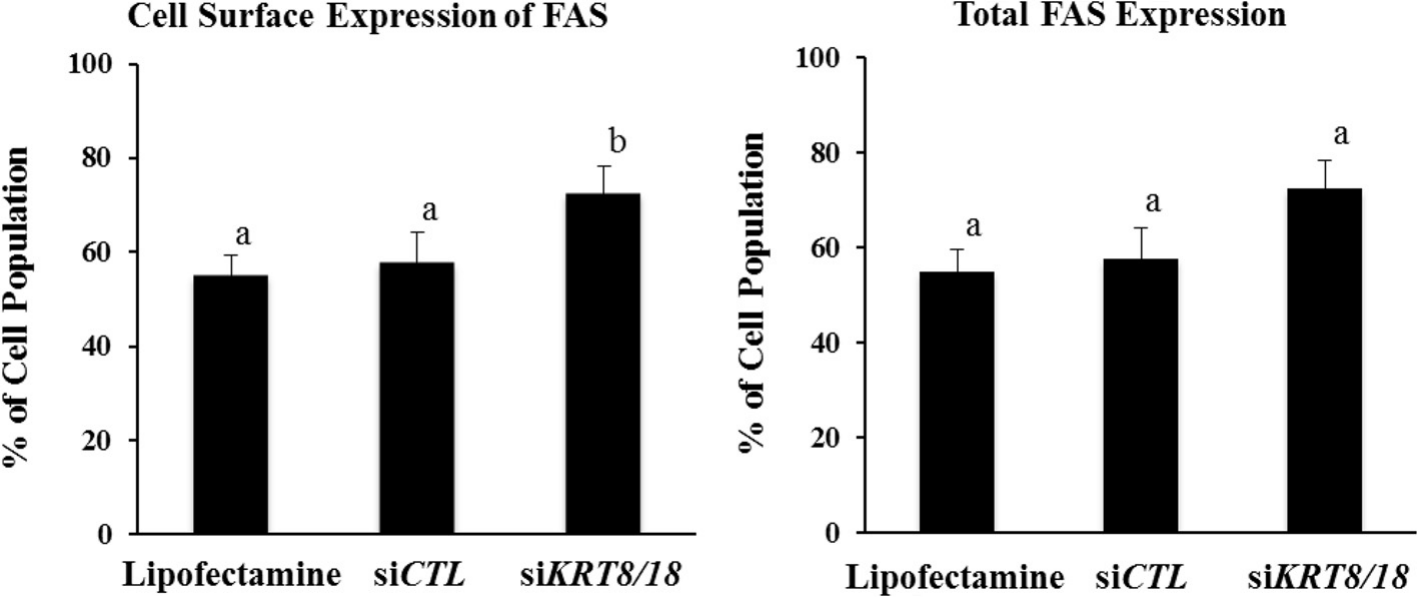
Measurement of the cell surface expression and total expression of FAS in KGN cells by flow cytometric analysis. The cells were analyzed following siRNA transfection with Lipofectamine™, si*CTL* or si*KRT8/18* (*n* = 6 experiments; different letters denote differences, *P* < 0.05)

Additional experiments revealed that siRNA-mediated reduction of K8/18 expression enhanced expression of FAS on the surface of KGN cells by 21 % as determined by flow cytometry (*P* < 0.05; Fig. 7). By comparison, no difference in total FAS expression (*P* > 0.05) was observed among the treatment groups (Fig. 7). Of note, Lipofectamine™ alone elevated the percentage of KGN cells that expressed FAS on the cell surface (58 %, Fig. 7) compared to that observed previously in non-treated controls (24 %, Fig. 2). However, the effect did not overall enhance the sensitivity of the cells to FasAb-induced apoptosis (*P* > 0.05 Fig. 6).

## Discussion

The current study is the first to demonstrate the expression of K8/18 filaments in KGN cells, a GCT-derived cell line, which has clinical relevance to GCT in particular, but also to general ovarian malignancy. Acknowledging that these intermediate filaments help to counteract physiological stressors [28, 39], we show that disrupting K8/18 filament expression (via siRNA) in KGN cells reverses resistance to FAS-mediated apoptosis by enhancing FAS expression on the cell surface

Keratin filaments are typically expressed by cells of epithelial origin [40]. Although the composition of keratin filaments can be quite diverse and is predicated by cell- and tissue-origin, previous reports document the assembly of K18-containing filaments within ovarian-derived tissues and tumors [26, 30, 38, 41]. Keratin filaments constitute one type of intermediate filament detectable within the ovary, particularly in granulosa cells of the follicle during stages of growth and atresia [30, 42], in luteal cells of the corpus luteum throughout the luteal phase [30, 38, 43], and in oocytes of fetal and adult ovaries [44, 45]. Other types of intermediate filaments expressed within cells of the ovary include vimentin and desmin [42, 46], which are of mesenchymal and myogenic origins, respectively [46]. Whether or not intermediate filaments other than K8/18 filaments impact FAS expression or FAS-mediated apoptosis is unknown.

FAS-mediated apoptosis is characterized by the localized secretion of Fas ligand, which then binds to FAS on the target cell, inducing oligomerization of the ligand-receptor complex. Oligomerization and internalization of the ligand-receptor complex triggers association with the cytoplasmic Fas-Associated Death Domain (FADD) protein [47]. Interestingly, FADD is the common point of convergence for both TNFR1-induced (via TRADD) and Fas ligand-induced pathways of apoptosis in cells [32]. In the current study, however, we focused only on the FAS-mediated pathway of cell death. Subsequent to activation, FADD facilitates the binding of pro-caspase 8 and the formation of the death inducing signaling complex (DISC). Assembly of the DISC results in cleavage of pro-caspase 8 to activate caspase 8, and initiates downstream events including the activation of caspase 3/7 enzymes that ultimately trigger apoptosis of the cell [48].

In the context of GC and GCT, the expression of FAS fundamentally determines the relative sensitivity of the cells to FAS-mediated apoptosis. Overexpression of wild-type, but not mutant *FOXL2*, in GC for example, upregulates FAS expression and augments Fas ligand-induced apoptosis [16]. Others report that granulosa and KGN cells normally resistant to Fas ligand-induced apoptosis become sensitized when pre-exposed to cytokines that up-regulate FAS expression, such as interferon gamma and tumor necrosis factor alpha [34, 37, 49]. In the present study we show for the first time that siRNA-mediated knockdown of K8/18 filaments in these cells also increases FAS expression and enhances FasAb-induced apoptosis. Consistent with the concept that FAS expression ultimately determines the sensitivity to FAS-mediated apoptosis, we also report that treatment of KGN cells with Lipofectamine™ increases trafficking of FAS to the cell surface and enhances responsiveness to FasAb, albeit to a lesser extent than the depletion of K8/18 filaments.

Inhibition of protein synthesis (via CHX) similarly enhances cell sensitivity to FasAb-induced apoptosis [36], but does so ostensibly by impairing the synthesis of cytoplasmic and labile, anti-apoptotic proteins rather than influencing FAS expresssion. In the current study, we observed increased apoptosis of KGN cells (i.e., increased caspase 3/7 activity and cleaved PARP expression) only after pretreating the cells with CHX for 2 h and then exposing them to FasAb (Figs. 3 and 4). Conversely, treatment with CHX or FasAb alone failed to induce apoptosis, and there was no effect of either treatment, alone or in combination, on the total expression of FAS or cFLIP (Fig. 4), a well-documented anti-apoptotic protein [50]. It is unclear why in the current study transient inhibition of protein synthesis failed to reduce cFLIP expression, yet augmented FasAb-induced apoptosis. Others have suggested that cFLIP plays an important role in preventing apoptosis in KGN cells [36, 51] and that CHX inhibits cFLIP expression [36, 52]. One explanation for the discrepancy in results between these previous reports and the current study might be the concentration of CHX used to inhibit protein synthesis (5-25 μg/mL in previous reports versus 0.25 μg/mL in this study, respectively). Isoforms of cFLIP also exist [53–55], which further complicates the interpretation of direct comparisons among studies. For these reasons, we suggest that additional investigation is needed to clarify the role of cFLIP as well as other potential anti-apoptotic molecules in KGN cells.

The activation of MAPK/ERK1/2 and PI3K/AKT signaling pathways have been implicated in the proliferation of KGN cells [56]. In previous work, we determined that inhibitors of these pro-survival signaling pathways prevent TGFα-induced phosphorylation of ERK1/2 and AKT in KGN cells [57], providing a potential role for TGFα in GCT initiation, progression and metastasis. However, inhibition of ERK1/2 phosphorylation has little to no effect on cell cycle progression or proliferation of KGN cells. Conversely, blocking AKT activation, or its downstream target mTOR, impairs cell cycle progression and prevent cell proliferation in cell culture [57] and in vivo models of GCT [58]. Thus, the AKT pathway appears more influential on the tumorigenic potential of KGN cells than the ERK1/2 pathway, and this is consistent with the identification of *AKT1* as a driver gene of GCTs [19]. In the present study, we observed that inhibiting either the ERK1/2 or AKT pathway (via PD98059 and Wortmannin, respectively) failed to augment the apoptotic effects of FasAb, as suggested by other models [59–61]. In the context of the present study, keratin filaments are known to regulate cell cycle progression [62, 63]. Therefore, we speculate that these filaments have the potential to modulate the effects of cell signaling inhibitors on KGN cell proliferation and apoptotic sensitivity.

The concept that keratin filaments influence the trafficking of cytokine receptors, as demonstrated in the current study, is a relatively new and unique attribute ascribed to intermediate filaments. Inhibition of keratin filament expression deems epithelial cells and carcinoma cell lines more sensitive to FAS-mediated apoptosis via a receptor mechanism [64, 65]. Additionally, expression of keratin filaments within a lymph node metastatic carcinoma cell line impairs human leukocyte antigen (HLA) class I receptor expression, enabling the cells to avoid destruction by CD8+ cytotoxic lymphocytes [66]. Our observations in which siRNA-mediated knockdown of K8/18 filaments in KGN cells increased FAS expression and enhanced sensitivity to FasAb-induced apoptosis is consistent with evidence that shows knockdown of *KRT18* increases FAS expression in both non-tumorigenic and tumorigenic cells [28, 33]. The abundance of keratin filaments in tumorigenic cells and their influence on metastatic potential has generated considerable interest among oncologists in recent years [67–69]. The current thinking is that keratin filaments facilitate migration of cancer cells in a manner separate from epithelial-mesenchymal transitions. Yet, surprisingly, this role of keratin filaments in cancer progression also renders some cell-types more sensitive to cisplatin therapy [33]. Of relevance to GCT, Woods and co-workers determined that KGN cells are resistant to cisplatin and tumor necrosis factor-related apoptosis inducing ligand [35, 70]. Unlike many other types of cancer cells, however, the KGN cells remain insensitive to these chemotherapeutic agents even after inhibition of NF-κB [35]. In the context of the current study, it is evident that targeting keratin filament expression in GCT and elevating FAS expression offers an alternative, clinical approach to surgical resection or platinum-based chemotherapy. However, therapeutic interventions that impair keratin filament expression/organization and enhance the vulnerability of epithelial cancers to apoptosis might also have the undesirable effect of augmenting tumorigenic potential [33].

## Conclusions

In conclusion, the abundance of keratin filaments in KGN cells provides a clinically-relevant mechanism of resistance to FAS-mediated apoptosis by impairing FAS trafficking and possibly activating downstream anti-apoptotic signaling molecules. Here, we provide evidence that decreased expression of FAS at the cell surface, and the presence/activation of labile protein(s) linked to keratin filament expression, provide the means of apoptotic resistance. The existence of keratin filaments in KGN cells and their role in apoptotic resistance provides insight on therapeutic strategies and tumorigenicity of cancers of ovarian origin, specifically GCT and OSE tumors.

## Abbreviations

CASP8: caspase 8
CFLAR: CASP8 and FADD-like apoptosis regulator
CHX: cycloheximide
DAPI: 4′,6-diamidino-2-phenylindole
DISC: death-inducing signaling complex
FAS: Fas cell surface death receptor (also known as TNFRSF6)
FasAb: FAS-activating antibody
FASL: FAS ligand
FITC: Fluorescein isothiocyanate
FSH: follicle-stimulating hormone
GCT: granulosa cell tumor
K: keratin (protein)
*KRT*: keratin (DNA/RNA)
MEK1/2: Mitogen-activated protein kinase kinase 1 and 2
NGS: normal goat serum
OSE: ovarian surface epithelium
PARP: Poly (ADP-ribose) polymerase
PI3K: Phosphatidylinositol-4,5-bisphosphate 3-kinase
siRNA: short interfering RNA
TLR: Toll-like receptor
TNFRSF1: Tumor necrosis factor receptor 1
TRAIL: Tumor necrosis factor (TNF)-related apoptosis-inducing ligand
